# Photocatalytic, antimicrobial and antibiofilm activities of MgFe_2_O_4_ magnetic nanoparticles

**DOI:** 10.1038/s41598-024-62868-5

**Published:** 2024-06-05

**Authors:** Ahmed M. El-Khawaga, Mohamed Ayman, Omar Hafez, Rasha E. Shalaby

**Affiliations:** 1 Department of Basic Medical Sciences, Faculty of Medicine, Galala University, New Galala City, Suez, Egypt; 2 Department of Microbiology and Immunology, Faculty of Medicine, Tanta University, Tanta, Egypt

**Keywords:** Antimicrobial activity, Antibiofilm activity, Photocatalysis, Magnetic nanoparticles, MgFe_2_O_4_ nanoparticles, Carbol fuchsin, Environmental sciences, Environmental biotechnology, Nanobiotechnology, Nanoparticles

## Abstract

This study reports the antibacterial and antibiofilm activities of Magnesium ferrite nanoparticles (MgFe_2_O_4_) against gram-positive and gram-negative bacteria. The photocatalytic degradation of Carbol Fuchsin (CF) dye (a class of dyestuffs that are resistant to biodegradation) under the influence of UV-light irradiation is also studied. The crystalline magnesium ferrite (MgFe_2_O_4_) nanoparticles were synthesized using the co-precipitation method. The morphology of the resulting nanocomposite was examined using scanning electron microscopy (SEM), while transmission electron microscopy (TEM) was employed for further characterization of particle morphology and size. Fourier transform infrared (FTIR) spectroscopy and X-ray diffraction (XRD) were utilized to analyze the crystalline structure, chemical composition, and surface area, respectively. Optical properties were evaluated using UV–Vis spectroscopy. The UV-assisted photocatalytic performance of MgFe_2_O_4_ nanoparticles was assessed by studying the decolorization of Carbol fuchsin (CF) azo dye. The crystallite size of the MgFe_2_O_4_ nanoparticles at the (311) plane, the most prominent peak, was determined to be 28.5 nm. The photocatalytic degradation of 10 ppm CF using 15 mg of MgFe_2_O_4_ nanoparticles resulted in a significant 96% reduction after 135 min at ambient temperature (25 °C) and a pH value of 9. Additionally, MgFe_2_O_4_ nanoparticles exhibited potent antibacterial activity against *E. coli* and *S. aureus* in a dose dependent manner with maximum utilized concentration of 30 µg/ml. Specifically, MgFe_2_O_4_ nanoparticles demonstrated substantial antibacterial activity via disk diffusion and microbroth dilution tests with zones of inhibition and minimum inhibitory concentrations (MIC) for *E. coli* (26.0 mm, 1.25 µg/ml) *and S. aureus* (23.0 mm, 2.5 µg/ml), respectively. Moreover, 10.0 µg/ml of MgFe_2_O_4_ nanoparticles elicited marked percent reduction in biofilm formation by *E. coli* (89%) followed by *S. aureus* (78.5%) after treatment. In conclusion, MgFe_2_O_4_ nanoparticles demonstrated efficient dye removal capabilities along with significant antimicrobial and antibiofilm activity against gram-positive and gram-negative bacterial strains suggesting their potential as promising antimicrobial and detoxifying agents.

## Introduction

Water pollution has become a global threat to mankind due to accelerated pace of global industrialization^1–3^. According to sustainable development goal (SDG) 6.3, the provision of uncontaminated water and the development of safe and sustainable water purification methods are of paramount importance from environmental, industrial, and societal, and perspectives^4^. It is estimated that poor drinking water quality contributes to 80% of world’s diseases and 50% of child deaths^5^. Given the extensive contamination of water resources, there has been increasing endeavors towards developing safe and effective water treatment strategies over the recent decades^6,7^. According to the World Bank estimates, approximately 17–20% of global pollutants can be attributed to the textile industry^8^. The persistent nature of these pollutants stems from the stability of the water-soluble compounds used in textile industry such as dyes, resulting in prolonged environmental impacts. Carbol fuchsin (CF), a cationic organic dye that is used as a biological stain and in addition to other applications^9^. As per the regulations outlined in 29 CFR 1910.1200 by the Occupational Safety and Health Administration (OSHA), CF is categorized as a hazardous substance^10^. Furthermore, Carbol fuchsin has the potential to cause various health hazards, including chemical burns. Considering the available literature highlighting its potential environmental accumulation, especially in water, and its hazardous effects, novel methods for its safe and efficient removal should be considered^11^.

Traditional approaches to water treatment like coagulation, flocculation, sedimentation, and filtration, have been employed^12,13^. However, these methods often fall short in terms of efficiency and cost-effectiveness. Photocatalytic decomposition process is one of the most applicable processes that have been utilized for industrial dyes degradation^14^. The utilization of nanomaterials in wastewater treatment for removal of harmful dyes has been identified as a green and environmentally friendly approach which presents significant opportunities to revolutionize the approach for the removal of toxic dyes, particularly, magnetic nanoparticles^15–22^. Magnetic nanoparticles possess exceptional properties such as high surface area to bulk volume ratios, low toxicity, increased activity, thermal stability, adaptability for surface modifications, and efficient dispersibility^23^.

In recent years, the problem of multidrug-resistance and extensive drug-resistance to antibiotics has surged to a critical level that requires urgent intervention. The World Health Organization (WHO) has alarmingly warned that a "post-antibiotic era" has arrived in which previously treatable infections can become life-threatening due to antimicrobial resistance (AMR). This poses unimaginable risks to human and animal health in the twenty-first century. Additionally, there is decline in the development of new antibiotics due to elevated costs of development coupled with low return on investment^24^. According to a global report on tackling drug-resistant infections, AMR is expected to be the cause of death of one person every three seconds by 2050, if no novel therapeutic modalities were developed to combat AMR^25^. Inorganic nanoparticles with magnetic properties offer a promising solution for fighting antibiotic-resistant bacteria that don’t rely on targeted blocking of specific antibiotic resistance pathways. Instead, these nanoparticles rely on antimicrobial mechanisms such as reactive oxygen species (ROS) generation, delivery of metal ions, and magnetic hyperthermia to which the bacteria have not been able to evolve resistance mechanisms^26,27^.

Ferrites are chemical compounds with magnetic characteristics that present as powder or ceramic substances. Their ferrimagnetic characteristics are primarily attributed to iron oxides, specifically Fe_2_O_3_ and FeO, which can be partly replaced by other transition metal oxides^28^. Magnesium ferrite (MgFe_2_O_4_) is one of the most important ferrites. It has a cubic structure of normal spinel-type and is a soft magnetic *n*-type semiconducting material. It is utilized in various fields such as magnetic technologies, sensors, adsorption, and heterogeneous catalysis^29^. In recent years, nanostructures of magnetic materials have garnered increasing interest because of their exceptional material characteristics, which are particularly different from those of bulk materials^30^.

There is a growing interest in examining the antimicrobial properties of MgFe_2_O_4_ nanoparticles (NPs) that is driven by the escalating antimicrobial resistance towards conventional antibiotics and the potentially promising antimicrobial properties of MgFe_2_O_4_ magnetic nanoparticles^31,32^.

The current study has two key objectives. The first is to conduct an in-depth analysis of the efficacy of magnesium ferrite nanoparticles in water treatment, with a particular focus on their adsorption capabilities and photocatalytic properties. The second objective is to investigate their antimicrobial and antibiofilm activities against certain gram-positive and gram-negative bacterial strains. Through this comprehensive investigation, we aim to contribute to the development of more effective, sustainable, and safe solutions for water treatment and antimicrobial applications, thereby addressing the pressing global issue of water contamination and antibiotic resistance.

## Materials and methods

### Materials

The materials utilized in this study were ferric chloride hexahydrate (FeCl_3_·6H_2_O) and magnesium chloride hexahydrate (MgCl_2_·6H_2_O). Carbol fuchsin (CF) was obtained from Sigma Aldrich; purity ≤ 100%). All the chemicals were of analytical grade and were utilized without additional refinement. Distilled water was used in nanoparticle preparation experiments.

### Synthesis of Mg Fe_2_O_4_ NPs

MgFe_2_O_4_ NPs were synthesized via the co-precipitation technique^33^. The desired chemical composition is achieved by combining equimolar quantities of magnesium chloride (MgCl_2_·6H_2_O) and anhydrous ferric chloride (FeCl_3_) dissolved in distilled water. The neutralization process is performed using a 1 M solution of sodium hydroxide (NaOH), with the reaction temperature held at 60 °C. The pH of the solution is maintained at 8 and stirring continues for duration of 2 h. The resulting precipitate is then washed with distilled water until it attains a state devoid of impurities. Subsequently, the product is subjected to drying at 100 °C to remove any remaining water content^34^.

### Characterization of MgFe_2_O_4_ NPs

The composition of functional groups and surface characteristics of MgFe_2_O_4_ NPs were elucidated through FT-IR spectroscopy (JASCO FT-IR 3600 Infrared spectrometer). The samples under study were analyzed using the KBr disc method, with spectra recorded in the range of 400–4000 cm^−1^. X-ray diffraction (XRD) analysis was then conducted on the synthesized Mg-F NPs using a Shimadzu XRD-6000 apparatus (SSI, Japan) to evaluate crystallinity, phase composition, crystallite size, and lattice properties. The X-ray diffraction patterns were generated by measuring the intensity of diffracted X-rays as a function of the diffracted angle (2θ). The average crystallite size was determined using the Williamson–Hall (W–H) method, as described by (Eq. 1)^35^.

1 $$D = 0.9\lambda / \beta \cos \theta$$

In this context, the symbol (λ) signifies the wavelength of the employed radiation, whereas (β) and (θ) represent the full width at half-maximum (FWHM) and the angle corresponding to the peak with the highest intensity, respectively. The dimensions and morphology of the synthesized MgFe_2_O_4_ nanoparticles (NPs) were analyzed using a High-Resolution Transmission Electron Microscope (HRTEM, JEM2100, Jeol, Japan). The surface characteristics and grain size of these MgFe_2_O_4_ NPs were examined using Scanning Electron Microscopy (SEM, ZEISS, EVO-MA10, Germany). Additionally, an Energy Dispersive X-ray (EDX) analysis was conducted with a BRUKER Nano GmbH instrument (Model D-12489, 410-M, Germany) to determine the elemental composition, purity, and relative proportions of each metal within the structured MgFe_2_O_4_ NPs.

### Antimicrobial activity testing of MgFe_2_O_4_ NPs

For antibacterial activity testing, gram-positive *Staphylococcus aureus* (ATCC 25923) and gram-negative *E. coli* (ATCC 25922) were selected as the target organisms to model two etiological agents of various infectious diseases in humans. The antimicrobial efficacy of the prepared Mg Fe_2_O_4_ NPs was qualitatively assessed using agar-disc diffusion and quantitatively using microbroth dilution methods^36–38^. Bacterial suspensions of 1.5 × 10^8^ CFU mL^−1^ (0.5 McFarland density) obtained from 18 to 24 h bacterial cultures developed on Trypticase Soy agar (TSA) were used. Three Mg Fe_2_O_4_ NPs concentrations (10, 20 and 30 mg/ml) were prepared in DMSO and dispensed by sonication. Sterile filter paper discs (6.0-mm) were saturated by the Mg Fe_2_O_4_ NPs solution and placed on the culture and incubated at 37 °C for 24 h after which the zones of inhibition were recorded. Sterile filter paper was saturated with DMSO and used as control. For performance comparison, conventional antibiotic disc containing Gentamycin (CN) (Oxoid) at a concentration of 10 μg and with a diameter of 6.0 mm was employed^39^.

For quantitative determination of the minimum inhibitory concentration (MIC) of MgFe_2_O_4_ NPs, Macrobroth dilution technique was performed in test tubes containing Muller Hinton broth (MHB) of 2 mL total volume according to previously reported methods^37^. Two bacterial suspensions of gram-positive *Staphylococcus aureus* (ATCC 25923) and gram-negative *E. coli* (ATCC 25922) were collected after 24 h culture on Nutrient broth and adjusted to 0.5 McFarland standard turbidity (1.5 × 10^−8^ CFU/mL). Different concentrations of Mg Fe_2_O_4_ NPs (0.025, 0.05, 0.1 and 0.2) were tested. A positive control tube containing uninoculated broth with nanoparticles and a negative control tube containing inoculated broth without nanoparticles were included. The MIC values were determined by Multi Mode Microplate reader (Hidex Sense) spectrophotometer after 24 h incubation at 36.0 ± 1.0 °C as the lowest concentration at which no visible turbidity could be detected^40^.

### Antibiofilm activity testing of MgFe_2_O_4_ NPs

A qualitative assessment of the antibiofilm activity of MgFe_2_O_4_ NPs was conducted by the tube adherence test according to the method outlined by a previous report^41^. Antibiofilm biofilm activity in the presence and absence of MgFe_2_O_4_ NPs at a concentration of 10.0 μg/mL. 5 mL of nutrient broth was introduced into both the treated and control tubes followed by the inoculation of the test bacteria (*S. aureus,* ATCC 25923) *and* (*E. coli,* ATCC 25922) at a concentration of 1.5 × 10^8^ CFU mL^−1^ (0.5 McFarland density). Subsequently, tubes were incubated at 37.0 ± 0.5 °C for 24 h. The media in both the control and treated tubes was removed then the tube was filled with Phosphate Buffer Saline (PBS) at pH 7.0. Bacterial cells adhering to the tube walls were dislodged by treating with 5 mL of 3.5% sodium acetate for approximately 20 min, followed by a thorough cleaning with deionized water. Biofilm formed on the inner walls of the tubes was stained with 20 mL of 0.15% Crystal Violet (CV) and subsequently rinsed with deionized water to remove excess CV. For semi-quantitative assessment of antibiofilm activity of NPs, 5 mL of absolute ethanol was introduced to dissolve the stained bacterial biofilm^38^. The optical density (O.D.) of the stained bacterial biofilm was measured using UV–Vis (Biochrom.

WPA Biowave III) spectrophotometer at 570.0 nm^39^. The percentage inhibition of bacterial biofilm formation in the presence of MgFe_2_O_4_ NPs was calculated using the following equation (Eq. 2)^40^:

2 $$Biofilm\; inhibition \% =[(O.D. Control\; sample - O.D. treated\; sample) / O.D. Control\; sample] x 100$$

### Photocatalytic degradation of Carbol fuchsin (CF) using MgFe_2_O_4_ NPs

A nanocomposite weighing 10 mg was introduced into a 50 ml aqueous solution containing Carbol fuchsin (CF) with an initial concentration (C_0_) of 10 mg/L. The mixture was stirred continuously at room temperature (25 °C) for 30 min in the absence of light to establish equilibrium between adsorption and desorption processes. Subsequently, a simulated UV light source, in the form of a UV lamp, was utilized to irradiate the solution containing the photocatalyst and CF. The UV lamp was positioned axially within a quartz immersion tube. At fixed time intervals during irradiation, a syringe equipped with a filter (pore size of 2.5 mm) was employed to extract a 1 ml sample of the CF suspension. The degradation rate of CF was determined by monitoring the change in CF concentration over the irradiation period using a UV–visible spectrophotometer (Agilent Technologies Cary 60 UV–visible) at a wavelength (λmax) of 545 nm. Deionized water served as the reference medium^41^.

### Ethics approval

This study complies with all relevant ethical regulations. The experimental protocol was approved by The Ethical Committee Review Board, Faculty of Medicine, Tanta University, Tanta, Egypt (approval code "36264PR481/12/23").

## Results and discussion

### Characterization of the synthesized MgFe_2_O_4_ NPs

#### XRD analysis of MgFe_2_O_4_ NPs

**Figure 1:**
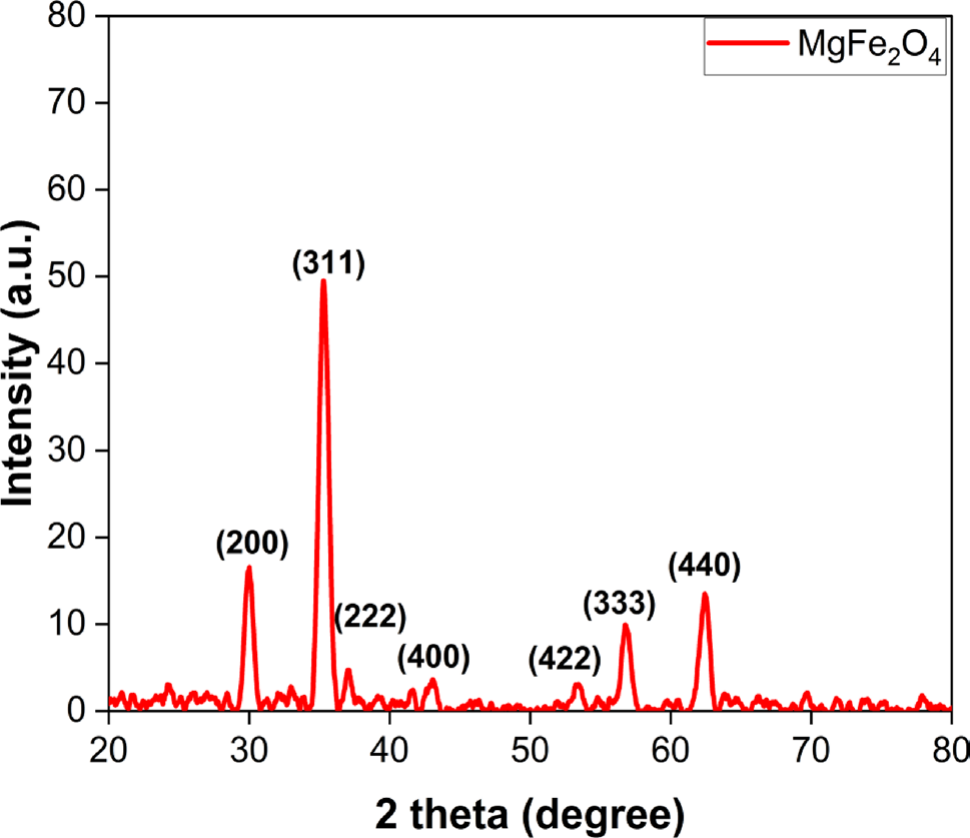
X-ray diffraction (XRD) patterns of the synthesized MgFe_2_O_4_ NPs.

Figure 1 illustrates the X-ray diffraction (XRD) patterns obtained for the synthesized MgFe_2_O_4_ nanocatalyst. The XRD patterns clearly reveal the presence of a cubic phase in the MgFe_2_O_4_ NPs, which can be matched to the standard JCPDS Card No. 22–1012(22–1042). In the diffraction patterns, each diffraction angle corresponds to a distinct peak, with notable peaks observed at approximately 2θ values of ~ 29.5°, 35.2°, 42.5°, 36.5°, 53.1°, 56.3°, and 62°^28^. The main diffraction peak at the (311) plane, with 2θ values around ~ 35.2°, is particularly noteworthy as it reflects the intensity of the cubic spinel ferrite. The average crystallite size was determined by analyzing the broadening of this diffraction peak, calculated using the Scherrer equation (Eq. 3)^29,30^:

3 $$D = K\lambda /\beta {\text{Cos}} \theta$$

In Eq. (3), (β) denotes the full width at half maximum (FWHM) of the diffraction peak, and K represents the shape factor. The shape factor, K, is approximately 0.9 and is equal to 0.15406, corresponding to the wavelength of CuK X-rays^26^. The crystallite size of the MgFe_2_O_4_ nanoparticles at the (311) plane, the most prominent peak, was determined to be 28.5 nm. It is noteworthy that the performance of nano photocatalytic substances is substantially influenced by their particle size.

#### Morphological analysis of Zn_0.5_Cu_0.5_Fe_2_O_4_ NPs

**Figure 2:**
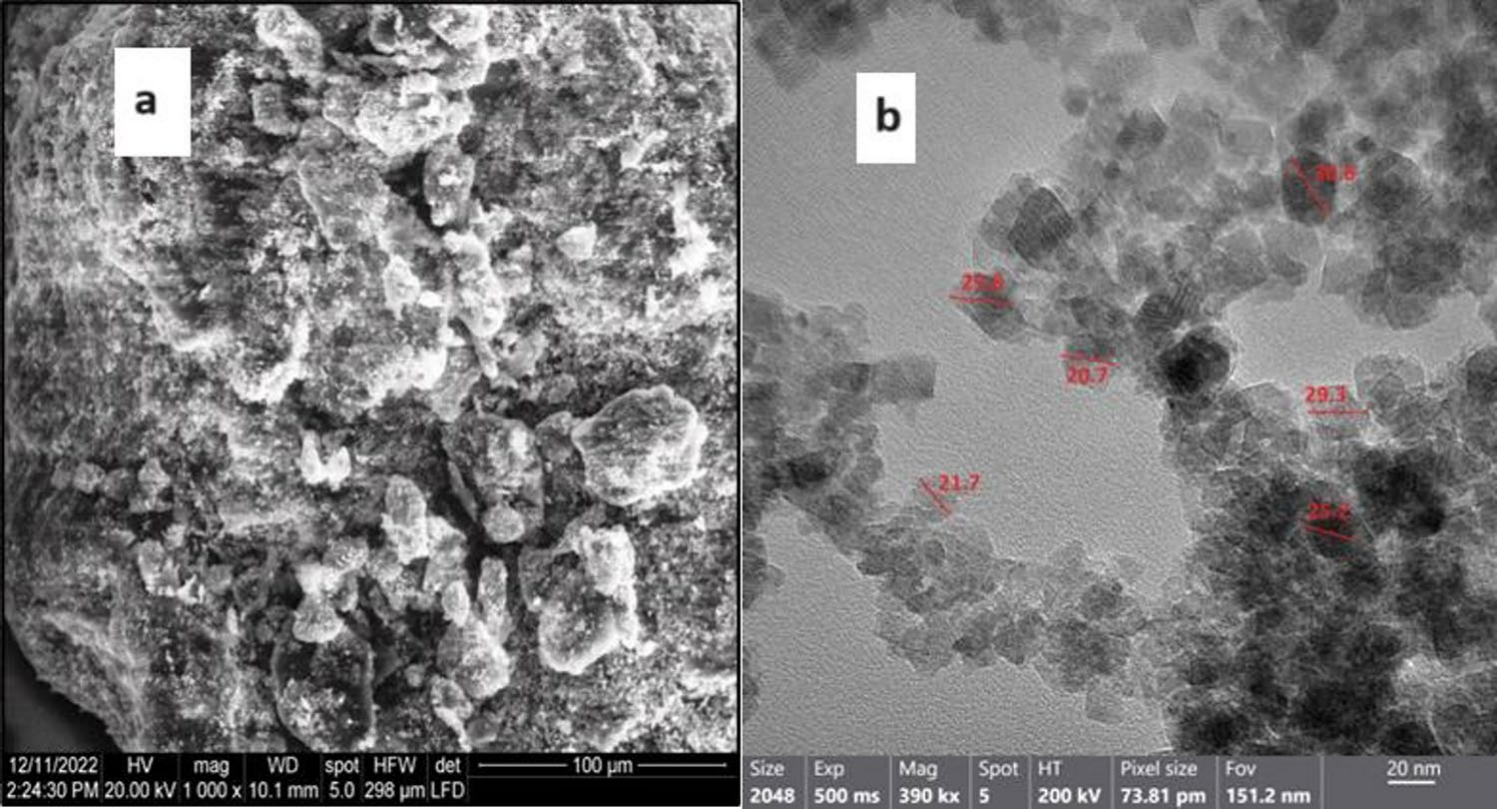
Electron microscopy images of the synthesized MgFe_2_O_4_ NPs. (**a**) SEM image of MgFe_2_O_4_ NPs. (**b**) HRTEM image of MgFe_2_O_4_ NPs.

The SEM image of MgFe_2_O_4_ nanoparticles, as depicted in Fig. 2a, indicates that the synthesized particles have a spherical morphology and aggregate readily; their grain sizes range between 25 and 35 nm. It is crucial to note that the particle sizes shown in the SEM pictures may not completely reflect the real properties of the sample. The restriction arises from the potential for surface charge of the samples during their interaction with the electron beam. Electrostatic charging may increase, particularly when no atmosphere exists, as the electron beam moves across non-conductive metal oxide surfaces. These conditions can result in a decrease in SEM magnification and level of detail. In contrast, HRTEM showed that the synthesized MgFe_2_O_4_ NPs appears as a semi-spherical shape and small sizes as shown in Fig. 2b. The detected sizes varied from 20.7 to 30.6 nm and the average particle size was determined to be 27.3 nm.

#### Fourier transform infrared spectroscopy (FT-IR) analysis of MgFe_2_O_4_ NPs

**Figure 3:**
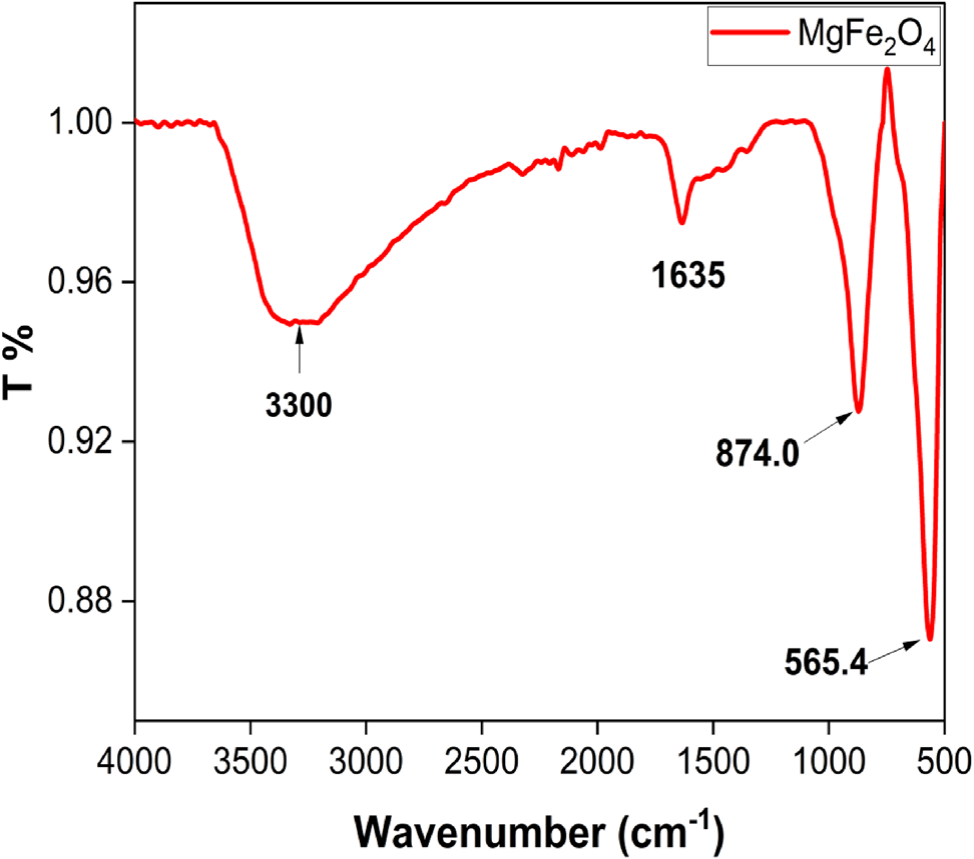
Fourier transform infrared spectroscopy (FT-IR) spectrum of the synthesized MgFe_2_O_4_ NPs.

The FT-IR spectrum of the synthesized MgFe_2_O_4_ nanoparticles (NPs), illustrated in Fig. 3, was obtained over a frequency range from 500 to 4000 cm^−1^. The spectra can be broadly classified into two main regions: the fingerprint region (400–1800 cm^−1^) and the lattice water H–O–H stretching band region (3200–3600 cm^−1^) ^31,32^. The characteristic absorption bands observed in the 550 to 700 cm^−1^ range are associated with the intrinsic vibrations of tetrahedral groups^28^. Furthermore, the absorption band at 1100 cm^−1^ can be ascribed to the stretching modes of metal–oxygen bonds (M=O), where molecules with a single terminal oxygen atom exhibit absorption^32^. Additionally, the peak observed at 1631 cm^-1^ corresponds to the bending vibration of O–H bonds in adsorbed water molecules present on the nanoparticle’s surface due to moisture adsorption. Furthermore, a peak at 3400 cm^-1^ is indicative of the stretching mode of adsorbed O–H groups.

#### UV–visible spectrophotometric and band gab analysis of MgFe_2_O_4_ NPs

**Figure 4:**
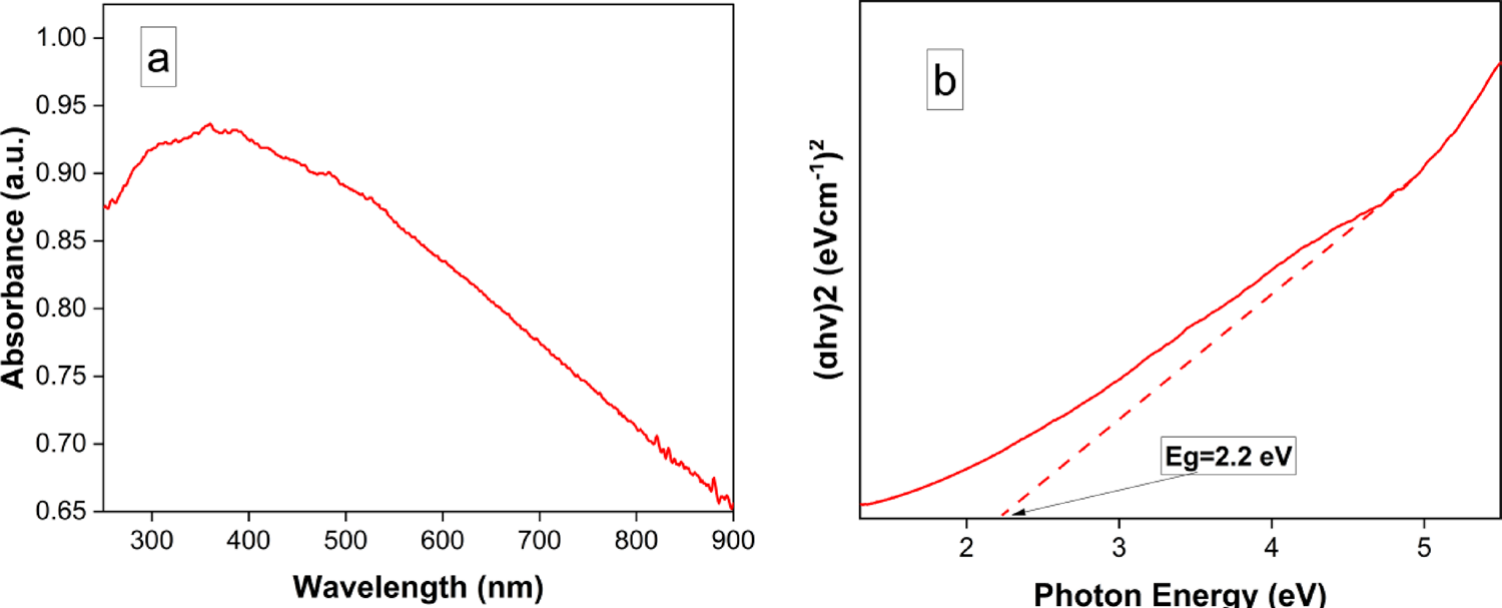
UV–visible spectrophotometric and band gab analysis of MgFe_2_O_4_ NPs. (**a**) UV–visible absorption spectrum of the MgFe_2_O_4_ nanoparticles. (**b**) The band gap energy of synthesized MgFe_2_O_4_ nanoparticles.

Optical absorption was used to evaluate the energy gap of the nanostructures shown in Fig. 4a. The MgFe_2_O_4_ NPs have low absorbance in the visible regions and high absorbance in the ultraviolet region^42^. The UV–visible spectrum clearly illustrates that MgFe_2_O_4_ NPs exhibit significant absorption characteristics within the wavelength range of 250–400 nm, which originates primarily from the absorption and scattering of light by the MgFe_2_O_4_ NPs. This heightened absorptivity is attributed to the involvement of the hybridized Fe–d orbital^43^. The band gap energy was determined using the absorption spectra and the Tauc relation, as shown in the inset of Fig. 4b. The calculated band gap energy was approximately 2.2 eV. It is worth noting that a higher band gap energy leads to a reduction in the recombination rate of electron–hole pairs, thereby enhancing the photocatalytic properties of the material.

### Antimicrobial and antibiofilm activities of the synthesized MgFe_2_O_4_ NPs

#### Antimicrobial activity of MgFe_2_O_4_ NPs

**Figure 5:**
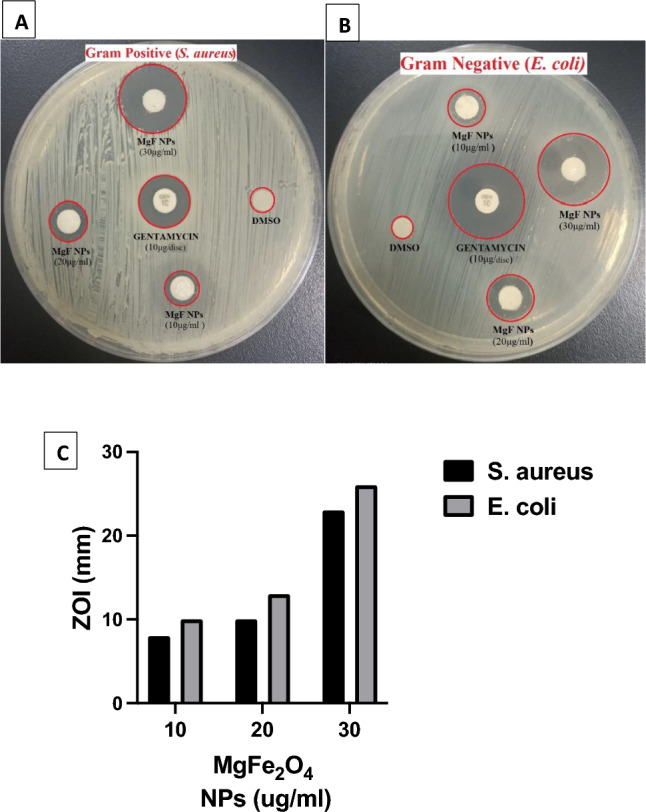
Antimicrobial activity of MgFe_2_O_4_ NPs by disk diffusion method. Antimicrobial activity of three concentrations of MgFe_2_O_4_ NPs_;_ 10, 20 and 30 µg/ml was tested against (**A**) *S. aureus* ATCC 52923, (**B**) *E. coli* ATCC 25922 and assessed by measuring ZOI (mm). DMSO was used as a negative control and Gentamicin (10ug/disc) was used as a positive control. (**C**) Quantitative analysis of antimicrobial activity of MgFe_2_O_4_ NPs.

The disk diffusion method was used to qualitatively assess the antimicrobial effectiveness of MgFe_2_O_4_ NPs against the gram-positive bacterium *S. aureus* (ATCC 25923) and the gram-negative bacterium *E. coli* (ATCC 25922). These particular strains were selected to model two clinically significant bacterial causes of several infectious diseases in humans. Additionally, their distinct cell wall structures might elicit varied reactions to the antimicrobial properties of MgFe_2_O_4_ NPs. As demonstrated in Fig. 5 and Table 1, 10ug/ml of MgFe_2_O_4_ NPs exhibited modest biocidal activity against both *S. aureus* and *E. coli* with zones of inhibition (ZOI) equivalent to 8 and 10 mm, respectively. However, a threefold increase in the concentration of MgFe_2_O_4_ NPs at 30ug/ml led to a proportional increase in biocidal activity against both *S. aureus* and *E. coli* with zones of inhibition equivalent to 23 and 26 mm, respectively. Enhanced growth inhibition effect was observed with the gram-negative bacterium *E. coli* compared to the gram-positive bacterium *S. aureus.* This differential inhibitory effect may be attributed to the distinct structure of their cell walls^44^. Further testing across a broader range of gram-positive and gram-negative bacterial strains is necessary to determine whether the observed antimicrobial activity of MgFe_2_O_4_ NPs is specific to certain species or broadly effective against both categories of bacteria. Consistent with the previous qualitative data, the quantitative antimicrobial assay via macrobroth dilution for MgFe_2_O_4_ NPs yielded an MIC value of 1.25 ug/ml for *E. coli*, which is half the MIC value yielded for *S. aureus*, at 2.5 ug/ml. This indicates that *E. coli* is more susceptible to MgFe_2_O_4_ NPs than *S. aureus* under the tested experimental conditions. The findings presented here align with other studies involving different metal nanoparticles, which have also demonstrated enhanced antimicrobial efficacy against *E. coli* relative to *S. aureus*^45–47^. Table 2 lists the reported antibacterial activity of different nanoparticles against *S. aureus* and *E.coli* from other studies, in comparison to our results.

**Table 1:** Antimicrobial activity assays of MgFe_2_O_4_ NPs by disk diffusion and macrobroth dilution methods.

Bacterial Strains	Inhibition Zone Diameter (mm)			MIC of MgFe_2_O_4_ NPs (µg/ml)	Percent reduction in biofilm formation (%)
	MgFe_2_O_4_ NPs, 10.0 µg/ml	MgFe_2_O_4_ NPs, 20.0 µg/ml	MgFe_2_O_4_ NPs, 30.0 µg/ml		
*S. aureus (ATCC 25923)*	8.0	10.0	23.0	2.50	82
*E. coli (ATCC 25922*)	10.0	13.0	26.0	1.250	92

MIC (Minimum inhibitory concentration).

#### Antibiofilm activity of MgFe_2_O_4_ NPs.

**Figure 6:**
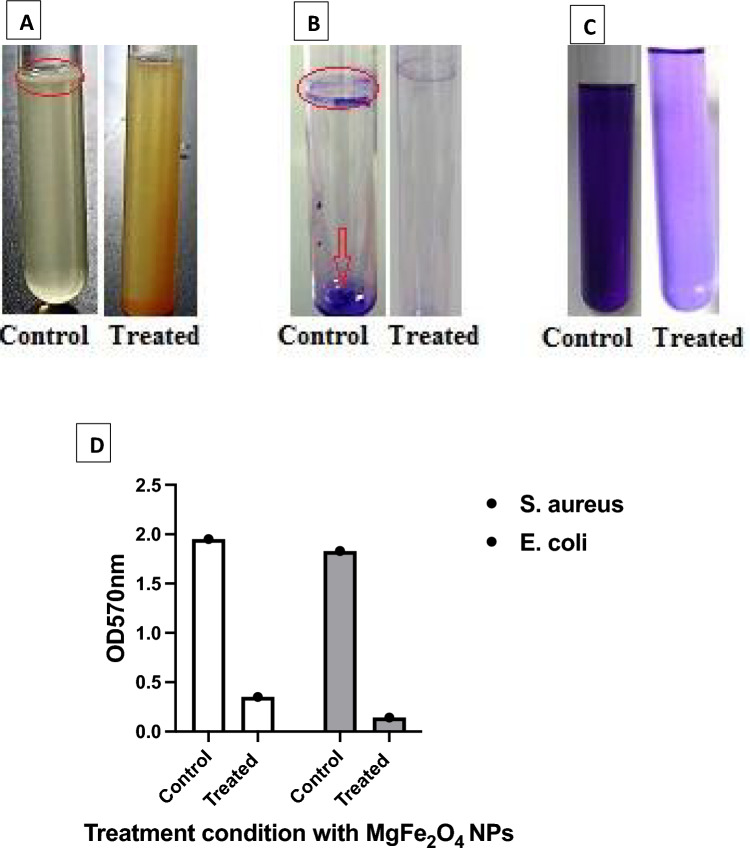
Antibiofilm activity of MgFe_2_O_4_ NPs *E. coli* using the tube adherence method. (**A**) Growth of bacterial cells and biofilm formation (rings) without treatment with MgFe_2_O_4_ NPs (left) and the inhibition of bacterial growth after treatment with MgFe_2_O_4_ NPs (right). (**B**) Staining of the adherent bacterial cells with crystal violet. (**C**) Removing and dissolving the adherent bacterial cells by ethanol for determination of quantitative biofilm inhibition (%). (**D**) Quantitative analysis of antibiofilm activity of MgFe_2_O_4_ NPs. The optical density (OD) of both the control sample (without MgFe_2_O_4_) and of the treated samples with 10ug/ml MgFe_2_O_4_ was measured at a wave length of 570 nm.

Several bacterial species of clinical importance are known to develop biofilm that is associated with increased antibiotic resistance^48,49^. Consequently, the design of novel antimicrobial agents that specifically target biofilms is crucial. In the current study, the antibiofilm activity of MgFe_2_O_4_ NPs was tested on two specific bacterial strains known for their biofilm-producing capabilities; *S. aureus* (ATCC 25923) *and E. coli* (ATCC 25922) by the tube adherence test^50,51^. Both *S. aureus* and *E. coli* inoculated in the absence of MgFe_2_O_4_ NPs developed a thick whitish yellow biofilm at the air–liquid interface as demonstrated for *E. coli* in Fig. 6A. This biofilm appeared as a blue ring after CV staining and was adherent to the tube wall (Fig. 6B). After dissolving the CV-stained blue ring with ethanol, a blue suspension was formed (Fig. 6C). Conversely, *S. aureus* and *E. coli* that were treated with MgFe_2_O_4_ NPs demonstrated marked inhibition of biofilm formation observed before and after CV staining (Fig. 6). The highest inhibition percentage was observed against *E. coli* (89%) followed by *S. aureus* (78.5%) after treatment with 10.0 µg/ml of MgFe_2_O_4_ nanoparticles (NPs), as shown in (Fig. 6D and Table 1). Elbasuney et al.^52^ reported that the addition of 10.0 µg/mL of Ag-HA nanocomposite resulted in the highest inhibition percentages of 96.09% and 95.60% against *S. aureus* and *E. coli*, respectively^52^. The differential percentage inhibition of biofilm formation by MgFe_2_O_4_ NPs for *E. coli* versus *S. aureus* may be due to multiple factors including differential antimicrobial activity, and distinct chemical interactions governing the antibiofilm properties of the nanocomposite on various bacterial species^44^. Table 2 listed the antibacterial activity of different nanoparticles against the selected bacterial strains compared to the obtained results from this study.

**Table 2:** Comparison of the antibacterial activity of different nanoparticles on *S. aureus* and *E. coli* by disk diffusion method.

Bacterial strains	Antibaterial agents	ZOI (mm)	References
*Staphylococcus aureus*	NiFe_2_O	6.4	^53^
	CoFe_2_O_4_	16.0	^54^
	MgFe_2_O	15.0	^55^
	Co_0.8_Mg_0.2_Fe_2_O_4_	23.0	^55^
	Zn_0.75_Co_0.25_Fe_2_O_4_ NPs	15.0	^56^
	MgFe_2_O_4_	23.0	Present study
*Escherichia coli*	MgFe_2_O_4_	17.0	^55^
	Ag–MgFe_2_O_4_	16.0	^57^
	Zn_0.6_Mg_0.4_Fe_2_O_4_	11.3	^58^
	CA-MgFe_2_O_4_ NPs	16.0	^34^
	CoFe_2_O_4_/PANI	12.3	^59^
	MgFe_2_O_4_	26	Present study

#### Proposed mechanism of the antimicrobial effect of the synthesized MgFe_2_O_4_ NPs

**Figure 7:**
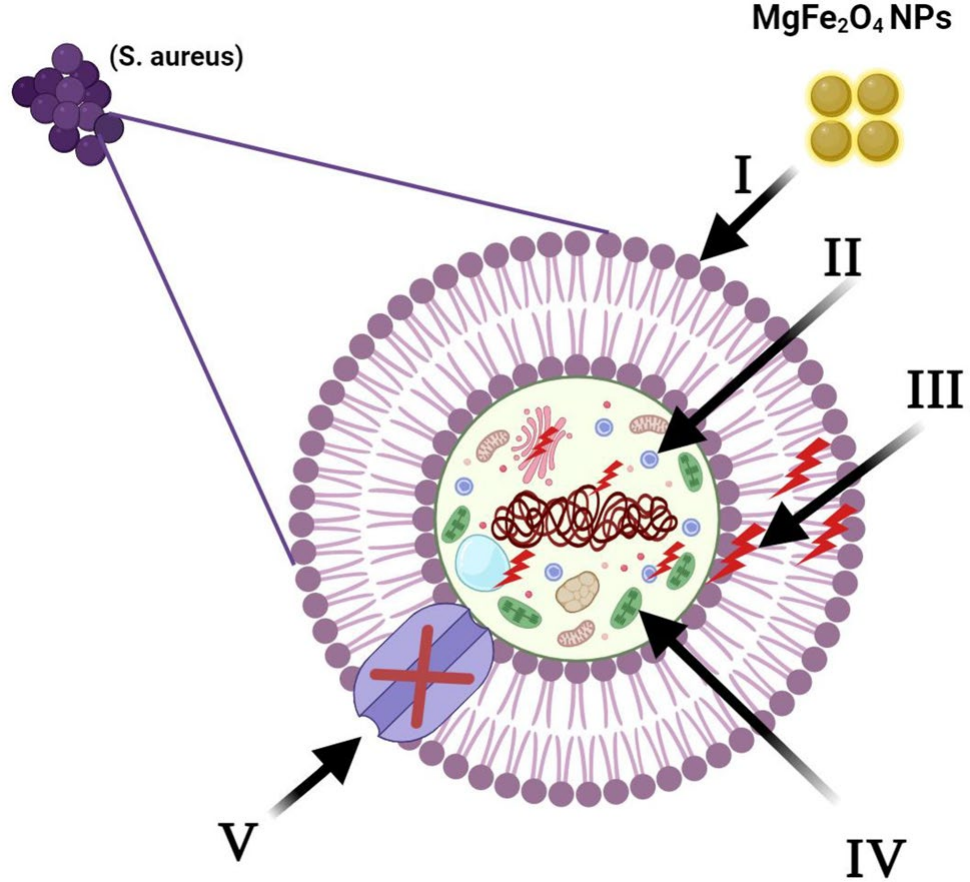
A model depicting the four main mechanisms underlying the antibacterial potential of the synthesized MgFe_2_O_4_ NPs. (**I**) MgFe_2_O_4_ NPs nanoparticles attach to and enfold the microbial cell surface, potentially via electrostatic interactions. (**II**) MgFe_2_O_4_ NPs penetrate the microbial cells and interact with cellular machinery components such as plasmid DNA, ribosomes, chromosomal DNA, and mesosomes, affecting their function. (**III**) MgFe_2_O_4_ induces the release and accumulation of ROS, leading to cellular damage. (**IV**) MgFe_2_O_4_ NPs modulate the cellular signal system and causing cell death. (V) Ultimately, the Mg-F nanoparticle composite impedes the bidirectional flow of ions across the microbial membrane.

An in-depth investigation is required to fully comprehend the antibacterial mechanism demonstrated by the synthesized  MgFe_2_O_4_ NPs. Figure 7 demonstrates a visual representation of the hypothesized antimicrobial action. It is understood that the activity of the synthesized MgFe_2_O_4_ NPs commences through their wrapping and adherence to the outer surface of microbial cells, resulting in membrane destruction and alteration of the transport potential. The MgFe_2_O_4_ NPs are posited to initiate contact by enclosing the outer layer of the microbial cells, leading to the breakdown of the cell membrane and alteration of the electrochemical gradient^60^. Following this initial interaction, the nanoparticles disseminate throughout the cell leading to cellular damage affecting bacterial genome, plasmid DNA, and other crucial organelles, primarily due to oxidative stress from reactive oxygen species (ROS) production. In the final stages, the nanocomposite impedes ion exchange with the across bacterial membranes^26^. However, morphological and topological features like shape, size, porosity, and surface roughness of NPs play a vital role in governing NP dissolution and antimicrobial efficacy^61^. Vishal Chaudhary et al. have proven that the size-dependent mechanism is the primary regulating element for the antibacterial efficacy and toxicity of AgNPs with diameters ranging from 1 to 10 nm^62^. Within this size range, Ag-NPs exhibit a higher dissolution rate than larger NPs. This is attributed to their increased surface-to-volume ratio, which aligns with the overall pattern of quantum size effects. Ag-NPs larger than 10 nm exhibit both ion-only and synergistic ion-particle processes, which ultimately act in a manner similar to their larger-scale counterparts. Furthermore, the structure of NPs also plays a crucial role in determining their antibacterial and dissolving performance. NPs with higher surface energy, such as nanoplates, dissolve faster than nanospheres. This is due to their smaller size and high-energy crystallographic aspects^26^.

### Photocatalytic degradation of Carbol fuchsin (CF) using MgFe_2_O_4_ NPs

**Figure 8:**
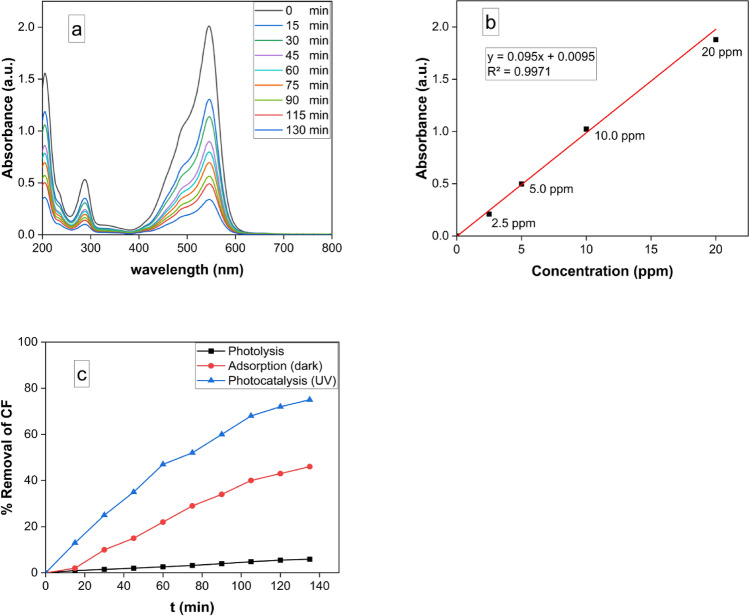
Photocatalytic degradation of Carbol fuchsin (CF) using MgFe_2_O_4_ NPs (**a**) Uv–Vis spectrum of CF after 15 min time interval using 10 mg of MgFe_2_O_4_ NPs under UV radiation. (**b**) Calibration curve of different concentrations of CF. (**c**) Percentage removal of CF photolysis is indicated by the black line, adsorption in dark by the red line, and UV photocatalysis by the blue line.

The removal of CF was monitored spectrophotometrically at its maximum absorbance wavelength, λmax = 545 nm^63^. Figure 8A clearly illustrates a progressive reduction in absorption peaks, indicative of CF dye photodegradation facilitated by the MgFe_2_O_4_ NPs photocatalyst, with increasing UV irradiation time. The calibration curve used for spectrophotometric analysis of CF samples was represented in Fig. 8B. As depicted in Fig. 8C, the photolytic degradation of CF amounted to only 7% after 135 min of UV irradiation. In contrast, the removal due to adsorption in the absence of light during the same timeframe was approximately 45.2%, as shown in Fig. 8C. On the other hand, the photocatalytic degradation of CF under UV light by MgFe_2_O_4_ nanocatalyst reached 76.0% after 135 min. This enhanced photocatalytic efficiency can be attributed to the presence of a metal–semiconductor heterojunction within the nanocomposite, which promotes effective charge separation and enhances light absorption.

#### Effect of pH on Removal of Carbol fuchsin (CF) by MgFe_2_O_4_ NPs

**Figure 9:**
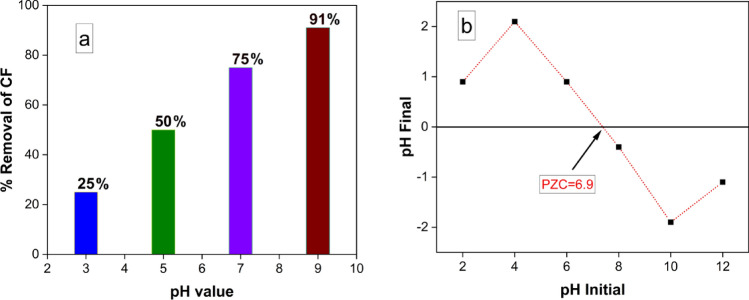
Effect of pH on removal of Carbol fuchsin (CF) by MgFe_2_O_4_ NPs. (**a**) Percentage removal of CF at different solution pH (5.0, 7.0 and 9.0). (**b**) Point of zero charge (PZC) of MgFe_2_O_4_ NPs at different pH values.

In the context of dye removal studies, an important variable to consider is the dependence on the solution's pH. We investigated the influence of initial pH values in a 90-min experiment conducted under specified conditions (10 mg of MgFe_2_O_4_ NPs, 50 ml of CF solution (10 mg/L) at 25 °C). Figure 9 presents a graphical representation depicting the removal of CF over time at different pH values (3.0, 5.0, 7.0, and 9.0). Notably, the maximum CF removal at equilibrium was observed at pH 9.0 as illustrated in Fig. 9a.

To determine the point of zero charge (PZC) of the MgFe_2_O_4_ nanocomposite, 0.01 g of MgFe_2_O_4_ NPs. was introduced into 50 mL of a 0.01 M NaCl solution. The pH of the solutions was adjusted using HCl or NaOH to achieve pH values of 2, 4, 6, 8, 10, and 12. Subsequently, the samples were stirred at 200 rpm for 48 h, and pH measurements were conducted after the magnetic separation of MgFe_2_O_4_ NPs.

The pH value at the Point of Zero Charge (PZC) was determined by plotting the final pH against the initial pH, as illustrated in Fig. 9b. The PZC pH was identified at pH = 6.9, where no significant difference existed between the final and initial pH values. This finding indicates that the surface charge of the MgFe_2_O_4_ NP photocatalyst is positive when the pH is below the PZC pH and negative when the pH exceeds the PZC threshold. Moreover, at the PZC pH, the surface charge of the photocatalyst becomes neutral, leading to minimal electrostatic interactions with ions such as CF ions. The calculated PZC pH value for MgFe_2_O_4_ NPs was found to be 6.9. The effective photocatalytic degradation of CF at pH 9.0, as shown in Fig. 9a, can be attributed to the negative net surface charge of MgFe_2_O_4_ NPs at this pH. This negative charge results in attraction between the negatively charged NPs and the positively charged CF, enhancing the degradation process. Conversely, the degradation of CF begins to decrease at pH 5.0, where the net surface charge of MgFe_2_O_4_ NPs becomes positive, leading to repulsion forces between the positive charge of CF and the positively charged surface of the NPs.

#### Effect of Initial Concentration of Carbol fuchsin (CF) on dye removal by MgFe_2_O_4_ NPs

**Figure 10:**
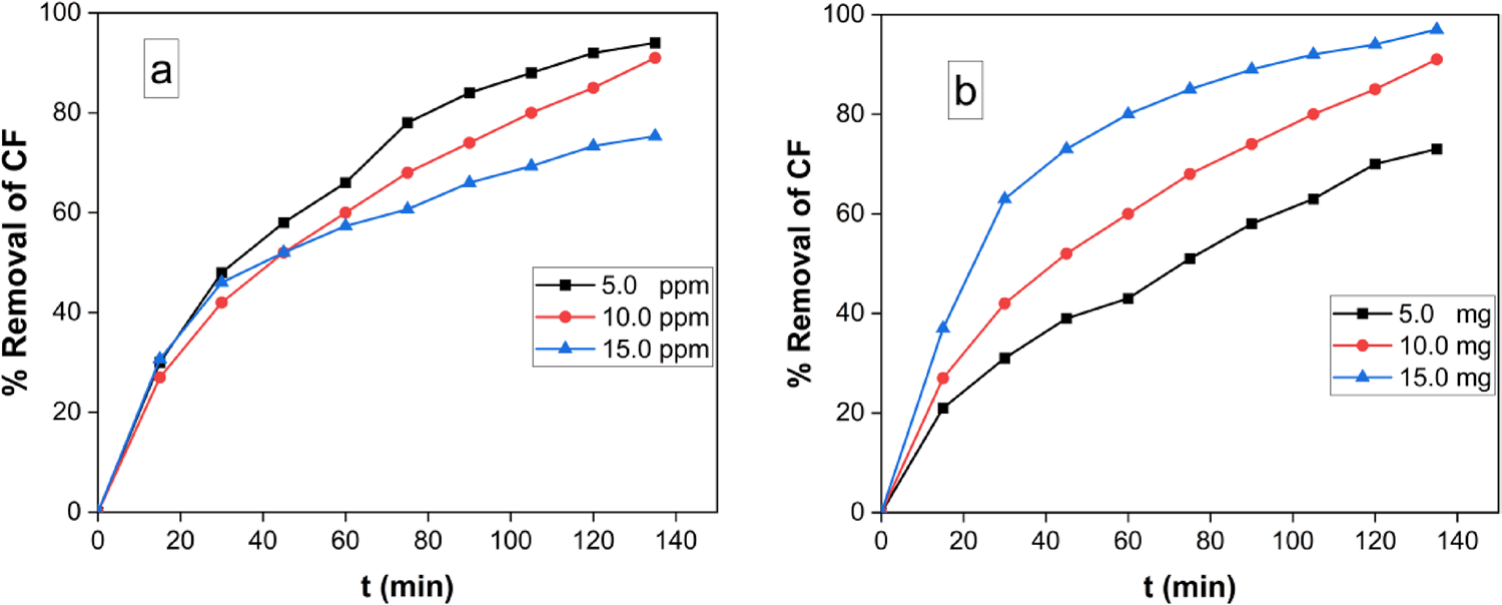
(**a**) The variation of percent removal as a function of contact time at different initial CF concentration (5, 10 and 15 ppm) at pH 9.0 and 10.0 mg MgFe_2_O_4_ NPs, (**b**) Effect of the photocatalyst dose on the Removal efficiency of CF (50 ml CF solution (10 mg/l), Temp. = 25 0C and pH 9.0).

We investigated the impact of varying the initial concentration of CF on its removal by MgFe_2_O_4_ nanoparticles (NPs) while maintaining constant reaction conditions. Figure 10a illustrates the variation in CF removal percentage over time for different initial CF concentrations (5.0, 10.0, and 15.0 mg/L). The findings indicate that as the initial CF concentration decreased from 5 to 15 mg/L, the removal efficiency increased. This suggests that even at low initial concentrations, CF can be effectively removed when exposed to UV light in the presence of the synthesized nanocomposite. This result could be explained by the Beer-Lambert Law, which states that an increment in initial dye concentration lowers the path length of photons exiting a solution. This reduces photon absorption by photocatalyst particles, drastically reducing the photocatalytic reaction rate^64^.

#### Effect of MgFe_2_O_4_ NPs dose on Carbol fuchsin (CF) degradation efficiency

The effect of varying doses of MgFe_2_O_4_ nanocomposite on the efficacy of CF removal under UV-light was studied by adjusting the quantity of the photocatalyst from 5 to 20 mg, with the CF concentration held constant at 10 mg/L, as illustrated in Fig. 10b. The results showed that the removal efficiency increased as the photocatalyst dose increased from 5 to 20 mg. The enhancement in removal efficiency with increasing photocatalyst quantity in the reaction could be attributed to the increased active area or active sites of the photocatalyst relative to the volume ratio of the CF solution^65^. Gao et al. reported similar behavior in dye degradation by using the cubic SrTiO_3_^66^.

#### Kinetic studies

The CF removal rate can be computed using the following equation (Eq. 4):

4 $$-ln Ct/CO = - Kt$$

**Figure 11:**
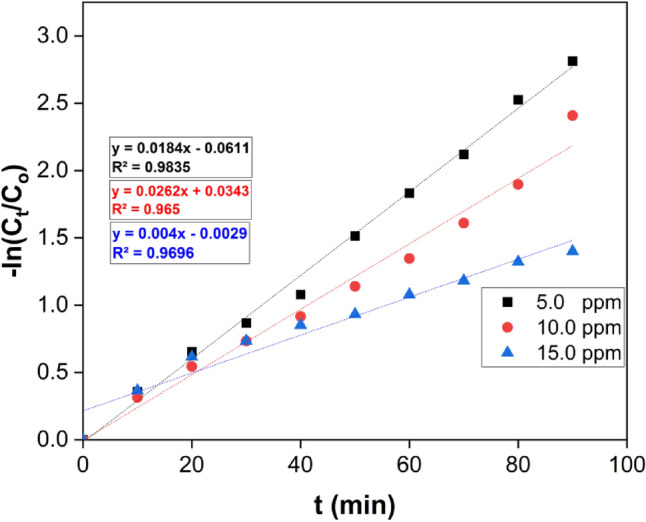
A linear fit, pseudo-first-order model data are reported in kinetic form for CF degradation under UV irradiation with beginning CF concentrations of 5, 10, and 15 ppm.

Here, C_t_ and C_o_ denote the remaining and initial concentrations of CF, respectively, while t represents the removal time, and k represents the removal rate constant. Figure 11 illustrates the relationship between -ln(C_t_/C_o_) and t. The findings indicated that the removal kinetics of the reaction adhered to pseudo-first-order rate kinetics. An increase in the initial concentration of CF led to a rise in the apparent pseudo-first-order rate constants, as seen in Fig. 11. The correlation between reaction rate constants and CF concentration is consistent with previous studies^67^.

#### Mechanism of photocatalysis of Carbol fuchsin (CF)

As reported in multiple studies^68–70^, the potential mechanism can be elucidated as follows: photodegradation mechanisms are influenced by variations in pH levels and involve the participation of hydroxyl radicals, oxidation via positive holes in the valence band, and reduction by electrons in the conduction band. Photocatalytic degradation is expected to happen in the presence of MgFe_2_O_4_ NPs when UV light promotes the formation of electron–hole pairs on the surface of MgFe_2_O_4_ NPs^71,72^. These holes can either react with -OH groups to produce hydroxyl radicals or oxidize the reactive CF to create a degradation product due to their oxidative potential^73,74^. Equations (5–8) summarize the reactions between CF and the photocatalyst used.

5 $${\text{MgFe}}_{{2}} {\text{O}}_{{4}} {\text{NPs }} + {\text{ hv}} \to {\text{MgFe}}_{{2}} {\text{O}}_{{4}} {\text{NPs}}\left( {{\text{ e}}^{ - }_{{{\text{CB}}}} + {\text{ h}}^{ + }_{{{\text{VB}}}} } \right)$$

6 $${\text{h}}^{ + }_{{{\text{VB}}}} + {\text{ MgFe}}_{{2}} {\text{O}}_{{4}} {\text{NPs}} \to {\text{MgFe}}_{{2}} {\text{O}}_{{4}} {\text{NPs}}^{ + } \left( {\text{oxidation of the compound}} \right)$$

(Or)

7 $${\text{h}}^{ + }_{{{\text{VB}}}} + {\text{ OH}}^{ - } \to {\text{OH}}$$

8 $${\text{OH}}^{.} + {\text{ CF}} \to \left( {\text{Degradation products}} \right)$$

**Figure 12:**
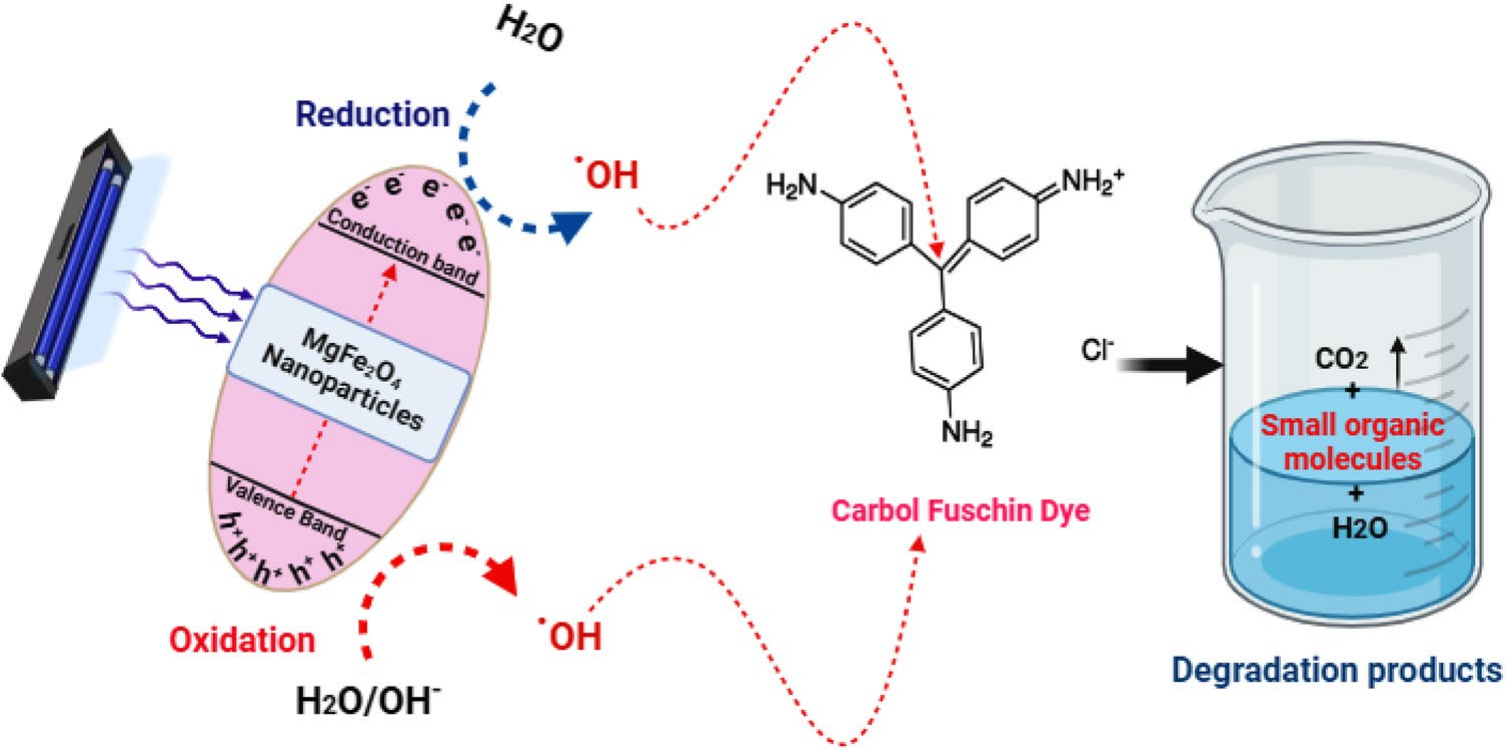
Potential photocatalytic reaction mechanism for CF photodegradation by MgFe_2_O_4_ NPs.

Figure 12 illustrates the suggested mechanism of the interaction between the produced MgFe_2_O_4_ nanoparticles and CF. When MgFe_2_O_4_ NPs are subjected to UV light, the excitation process generates charge carriers, initiating redox reactions. Consequently, the resultant free radicals, including OH· and O2·^−^, participate in the degradation of CF, resulting in the formation of smaller organic compounds. It is noteworthy that, as of the current juncture, A few reports have been published regarding the degradation products of CF. Therefore, further investigations employing analytical techniques such as high-performance liquid chromatography (HPLC) and gas chromatography-mass spectrometry (GC–MS) are imperative for a more comprehensive analysis of CF degradation products. Table 3 listed the photocatalytic degradation activities of the different nanoparticles compared to the obtained results from this study.

**Table 3:** Comparison of photocatalytic properties of different nanoparticles the literature.

Sl. no	Catalyst	Synthesis method	Dye	Irradiation time (min)	Degradation efficacy (%)	References
1	MgFe_2_O_4_	Microwave sintering	MB	180	26	^75^
2	MnFe_2_O_4_	Hydrothermal route	MB	1200	67.18	^75^
3	Fe_3_O_4_/FePC + H_2_O_2_	Organic–inorganic complexation		120	78	^76^
4	Fe_1.85_Cu_0.15_Zr_1.85_W_0.15_O_7_	Pechini method	CF	60	100	^77^
5	ZnFe_2_O_4_	Co-precipitation method	MB	60	29.7	^78^
6	Ni_x_Co_1–x_Fe_2_O_4_	Microwave-assisted green route	MB	120	99.76	^79^
7	BaSO_4_-NPs	one-step hydrothermal method	MB	150	89	^80^
8	Co_3_O_4_-NPs	Biosynthesis method	MB	105	89	^81^
9	MgFe_2_O_4_	Co-precipitation method	CF	135	96	Present study

## Conclusion

MgFe_2_O_4_ NPs were successfully synthesized through a co-precipitation methodology and subjected to comprehensive structural and optical characterization. The photocatalytic performance of the resultant MgFe_2_O_4_ nanocatalyst was evaluated using CF dye as the target pollutant. Additionally, a thorough exploration of various factors affecting the degradation efficacy, including pH levels during CF degradation, initial CF concentration, and the dosage of MgFe_2_O_4_ NPs as photocatalysts, was conducted. The MgFe_2_O_4_ NPs demonstrated notable photocatalytic efficiency in the removal of CF from aqueous solutions. Remarkably, under conditions of pH 9 and utilizing 15 mg of MgFe_2_O_4_ NPs, approximately 96% of a 10 ppm CF solution was effectively photodegraded after 135 min. In vitro assessments further corroborated the antimicrobial potential of MgFe_2_O_4_ NPs, as evidenced by zone of inhibition (ZOI) and minimum inhibitory concentration (MIC) results. Specifically, MgFe_2_O_4_ NPs exhibited significant antimicrobial activity against *E. coli* (ZOI: 26.0 mm, MIC: 1.25 µg/ml) and *S. aureus* (ZOI: 23.0 mm, MIC: 2.5 µg/ml). The synthesized MgFe_2_O_4_ nanocatalyst holds promise for applications in antimicrobial treatments and wastewater purification processes.

## Data Availability

Availability of data and materials: The data generated in the current study are available from the corresponding authors upon request.
