# Publisher Correction: Photocatalytic, antimicrobial and antibiofilm activities of MgFe_2_O_4_ magnetic nanoparticles

**DOI:** 10.1038/s41598-025-90562-7

**Published:** 2025-03-04

**Authors:** Ahmed M. El-Khawaga, Mohamed Ayman, Omar Hafez, Rasha E. Shalaby

**Affiliations:** 1 Department of Basic Medical Sciences, Faculty of Medicine, Galala University, New Galala City, Suez Egypt; 2 Department of Microbiology and Immunology, Faculty of Medicine, Tanta University, Tanta, Egypt

Correction to: *Scientific Reports* 10.1038/s41598-024-62868-5, published online 05 June 2024

The original version of this article omitted the ethics approval statement. This now reads:

“This study complies with all relevant ethical regulations. The experimental protocol was approved by The Ethical Committee Review Board, Faculty of Medicine, Tanta University, Tanta, Egypt (approval code "36264PR481/12/23").”

The original article has been corrected.

